# A case report of acute hyperlipidemic pancreatitis after blastocyst transfer and literature review

**DOI:** 10.3389/fmed.2026.1698359

**Published:** 2026-02-04

**Authors:** Minglin Liu, Hui Hu

**Affiliations:** Department of Obstetrics and Gynecology, The Second Affiliated Hospital of Nanchang University, Nanchang, China

**Keywords:** assisted reproductive technology, case report, diabetes, hypertriglyceridemic pancreatitis, non-alcoholic fatty liver disease

## Abstract

**Case:**

A 28-year-old woman with a history of type 1 diabetes and non-alcoholic fatty liver disease (NAFLD) but no other significant past medical history, abdominal pain and nausea and vomiting 9 days after blastocyst transfer. She was diagnosed with Hyperlipidemic pancreatitis (HTGP) associated with assisted reproduction following comprehensive diagnostic evaluations and admitted to the ICU of our hospital.

**Outcome:**

Upon admission, the patient was managed with nil per os (NPO), plasmapheresis, and supplemental insulin. Medications administered included fenofibrate, ulinastatin, latamoxef (moxalactam), and the traditional Chinese medicine Mirabilite (Glauber’s salt). Fresh frozen plasma (FFP) and albumin were also transfused. Two days later, the patient’s relevant indicators decreased significantly and symptoms improved. The patient was subsequently transferred to the general ward for continued treatment.

## Introduction

Acute pancreatitis is nowadays one of the most common diseases among gastroenterology disorders, being gallstones and alcohol the main etiologies (1). Severe hypertriglyceridemia is the third most common cause of acute pancreatitis. Hypertriglyceridemic pancreatitis after assisted reproduction is extremely rare, with only one case reported in the past 5 years (2). The diagnostic criteria for acute pancreatitis (AP) require: (1) typical epigastric pain consistent with pancreatitis, (2) serum lipase (or amylase) levels ≥3 times the upper limit of reference, and (3) radiological evidence of pancreatitis on contrast-enhanced computed tomography (CECT) or magnetic resonance imaging (MRI) (3). We introduce a 28-year-old woman who was diagnosed with hypertriglyceridemic pancreatitis 9 days after assisted reproductive technology (ART), with a triglyceride level reaching 155.00 mmol/L. In our study, we plan to conduct a systematic analysis of the patient’s medical examinations and test results to explore the possible association between assisted reproductive technology and acute pancreatitis, providing new insights for the diagnosis and treatment of assisted reproductive technology (ART) patients with underlying metabolic diseases such as diabetes and NAFLD.

## Case description

### Case presentation

A woman aged 28, who had previously given birth to a daughter, developed acute abdominal pain following a blastocyst transplantation on April 18, 2025. The oral medication for luteal phase support after embryo transfer is shown in Table 1.

**Table 1:** Medications used after blastocyst transplantation.

Medicine	Dosage	Frequency
Dydrogesterone	10 mg	BID
Estradiol valerate tablets	1 mg	QD
Progesterone vaginal sustained-release gel	90 mg	QD
Enoxaparin	0.2 mL	QD

On April 27, 2025, the patient suffered sudden onset of upper abdominal pain, which was well-localized and distending, accompanied by severe nausea and vomiting. The symptoms showed a progressive worsening, prompting the patient to refer herself to our hospital on the same afternoon on account of unbearable abdominal pain.

Prior to transfer to our institution, the patient’s serum *β*-HCG level measured at an external hospital was 61.8 IU/mL, with progesterone at 0.72 ng/mL. This level is strongly associated with a high risk of early pregnancy loss.

Past Medical History: The patient has a history of type 1 diabetes mellitus and non-alcoholic fatty liver disease. Currently, the patient is treating diabetes through non-pharmacological methods. The patient denies a history of hypertension, polycystic ovary syndrome, and pancreatitis.
Family History: Her mother suffered from diabetes. She denies any family history of hyperlipidemia.
Vital Signs and Anthropometric Measurements: On admission, the patient’s vital signs were recorded as follows: body temperature 36.5 °C, pulse rate 82 beats per minute, respiratory rate 18 breaths per minute, and blood pressure 134/80 mmHg. Height 161 cm, weight 63 kg, BMI 24.3 kg/m^2^.
General and Systemic Examination: No abnormalities.
Abdominal Examination: The abdomen was flat with no visible gastric or intestinal distension, peristaltic waves, abdominal wall varicosities, Cullen sign, or Grey-Turner sign. On palpation, the abdomen was soft, with tenderness elicited in the epigastric region and right hypochondrium. Murphy’s sign was suspiciously positive.

### Laboratory investigations-metabolic profile

Marked hypertriglyceridemia (155.00 mmol/L; reference <1.7 mmol/L) was accompanied by severe hypercholesterolemia (45.35 mmol/L; reference <5.18 mmol/L). HDL cholesterol (HDL-C) was within the normal range (1.88 mmol/L). Serum amylase was significantly elevated (156.50 U/L; reference 35–135 U/L), consistent with pancreatic involvement.

### Hematologic parameters

Marked leukocytosis (19.99 × 10^9/L; reference 3.50 × 10^9/L–9.50 × 10^9/L) was noted, accompanied by neutrophilia (17.49 × 10^9/L; reference 2.0 × 10^9/L–7.0 × 10^9/L) (Table 2).

**Table 2:** The test indicators on April 27th.

Laboratory indicators	The test results on April 27th	Reference
Aspartate aminotransferase	37 U/L	13-35 U/L
Alanine aminotransferase	55.7 U/L	7–40 U/L
Amylase	156.5 U/L	35-135 U/L
Total cholesterol	45.35 mmol/L	<5.18 mmol/L
Triglyceride	155 mmol/L	<1.7 mmol/L
High-density lipoprotein cholesterol	1.88 mmol/L	1.29–1.55 mmol/L
White blood cell count	19.99 × 10^9/L	3.50 × 10^9/L−9.50 × 10^9/L
Absolute neutrophil count	17.49 × 10^9/L	2 × 10^9/L−7 × 10^9/L
Serum albumin	21.4 g/L	40-55 g/L

### Abdominal CT imaging findings

1. Pancreatic morphology: The pancreas appears slightly enlarged with blurred margins, exhibiting heterogeneous parenchymal hypodensity.
2. Peripancreatic changes: Scattered, ill-defined patchy hypodense areas are seen to infiltrate the peripancreatic fat. Additionally, subtle thickening of the bilateral anterior renal fascia is noted.

### Transvaginal ultrasound findings (gynecological)

1. Uterine Cavity: No discernible intrauterine gestational sac is identified.
2. Adnexal Evaluation: Bilateral ovaries demonstrate normal sonographic morphology. No adnexal masses or cystic abnormalities are present.
3. Cul-de-sac Assessment: Absence of free pelvic fluid is noted. No hemoperitoneum is apparent.
4. Doppler Findings: Uterine and ovarian vascular flow patterns are normal. No aberrant vascular signals suggestive of ectopic implantation are detected.

### Abdominal ultrasonography findings: hepatic evaluation

**Figure 1:**
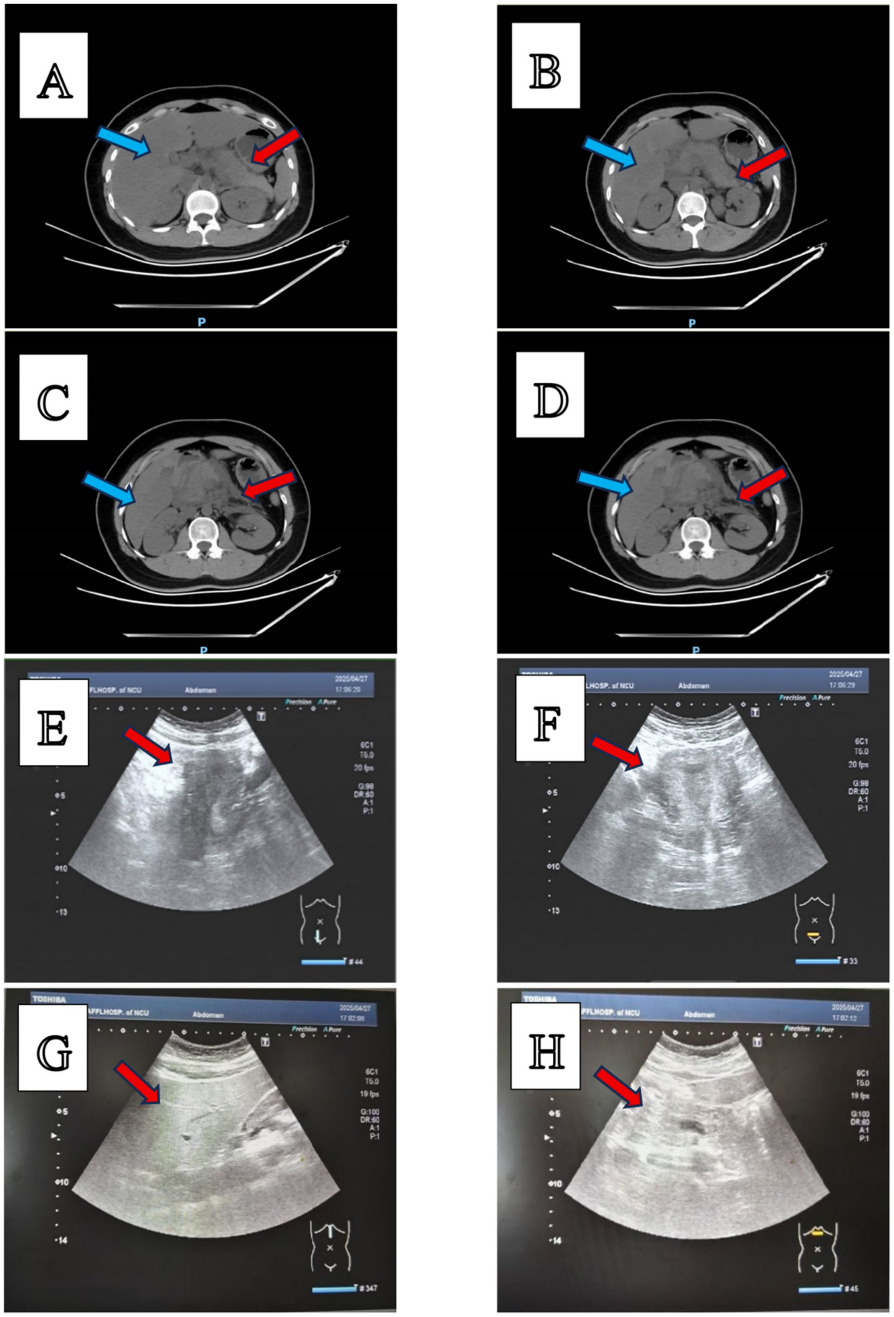
Imaging findings with parts **(A–H)**. **(A–D)** Abdominal contrast-enhanced computed tomography (CT) images. The red arrows indicate the pancreas, and the blue arrows indicate the liver. CT findings include: (1) Diffusely decreased hepatic parenchymal density with indistinct visualization of intrahepatic blood vessels; and (2) Mild enlargement of the pancreas, accompanied by heterogeneous reduction in parenchymal density, blurred margins, and scattered patchy hypodense lesions in the peripancreatic region. **(E,F)** Gynecological color Doppler ultrasound images. The arrows point at the uterus, in which no abnormal echoes were detected. **(G,H)** Abdominal ultrasound images. The arrows indicate the liver parenchyma, which exhibits diffusely increased fine-grained echogenicity; the hepatic capsule remains smooth.

1. Parenchymal Characteristics: The liver parenchyma exhibits diffusely increased echogenicity with a fine, homogeneous texture. Pronounced attenuation of ultrasound beam penetration is evident. Portal vein margins demonstrate relative sparing, with distinct margins.
2. Morphological Features: Hepatic contours remain smooth. Hepatic vascular architecture remains unremarkable. No focal parenchymal lesions are identified (Figure 1).

### Diagnostic and therapeutic workflow

Upon admission, the patient was diagnosed with the following comorbidities: Hyperlipidemic pancreatitis associated with assisted reproduction, non-alcoholic fatty liver disease (NAFLD), type 1 diabetes mellitus (T1DM), and electrolyte disturbances. Immediately, the patient was transferred to the intensive care unit (ICU) for comprehensive multidisciplinary management. Specifically, at approximately 21:00 on April 27 (post-admission), a jejunal feeding tube was placed for gastrointestinal decompression and to enable early enteral feeding. Concurrently, the patient was prescribed strict fasting, along with supportive interventions including intravenous fluid resuscitation. Insulin supplementation was administered at a daily dose of 8 units (QD). For pancreatitis control, ulinastatin was administered every 8 h (Q8H). To manage systemic inflammation, a combination of external mirabilite application and intravenous latamoxef infusion was implemented every 12 h (Q12H). To slow down the course of the hypercoagulable state of the blood, enoxaparin is injected subcutaneously every 12 h (Q12H) to prevent venous thromboembolism (VTE) (Table 3). Plasma exchange was performed at approximately 00:30 on April 28. Post-procedure, a follow-up lipid profile was obtained, which demonstrated a reduction in total lipid levels from 45.35 mmol/L (on April 27) to 22.66 mmol/L, and a decrease in triglyceride (TG) levels from 155.00 mmol/L (on April 27) to 86.76 mmol/L. On the morning of April 28, oral fenofibrate was initiated once daily (QD) for lipid-lowering therapy, and intravenous albumin supplementation was commenced. At noon on April 28, the patient received a transfusion of allogeneic B-type, Rh-positive fresh frozen plasma. The patient had a reexamination of blood lipid and blood routine on April 29th, and the results are as follows.

**Table 3:** Treatment after being transferred to our hospital.

Time	Treatment	Frequency
9 p.m. on April 27th, 2025	A jejunal feeding tube was placed	
	Fasting	
	Intravenous fluid resuscitation	
	Insulin	8U, QD
	Ulinastatin	Q8H
	Mirabilite	Q12H
	Latamoxef	Q12H
Half past midnight on April 28th, 2025	Plasma exchange	
On the morning of April 28th,2025	Fenofibrate	QD
	Albumin	
At noon on April 28th,2025	Fresh frozen plasma	

### Follow-up laboratory results and clinical progress (April 29, 2025)

#### Metabolic and nutritional parameters

Significant improvement was observed in the lipid profile: total cholesterol normalized to 4.17 mmol/L, and triglyceride levels decreased sharply to 8.69 mmol/L. Serum albumin levels also increased to 36.4 g/L.

#### Inflammatory and hematologic markers: marked inflammation with partial resolution

Leukocytosis exhibited a trend toward improvement, with the white blood cell (WBC) count decreasing from the admission baseline of 21.65 × 10^9/L to 11.17 × 10^9/L; the absolute neutrophil count (ANC) decreased to 9.52 × 10^9/L (Table 4).

**Table 4:** The test indicators on April 29th.

Laboratory indicators	The test results on April 29th.	Reference
Aspartate aminotransferase	28.10 U/L	13-35 U/L
Alanine aminotransferase	13.10 U/L	7-40 U/L
Total cholesterol	4.17 mmol/L	<5.18 mmol/L
Triglyceride	8.69 mmol/L	<1.7 mmol/L
High-density lipoprotein cholesterol	0.52 mmol/L	1.29–1.55 mmol/L
White blood cell count	11.17 × 10^9/L	3.50 × 10^9/L−9.50 × 10^9/L
Absolute neutrophil count	9.52 × 10^9/L	2 × 10^9/L−7 × 10^9/L
Platelet count	117 × 10^9/L	125 × 10^9/L−350 × 10^9/L
Glucose	10.87 mmol/L	3.9–6.1 mmol/L
Albumin	36.4 g/L	40–55 g/L

**Figure 2:**
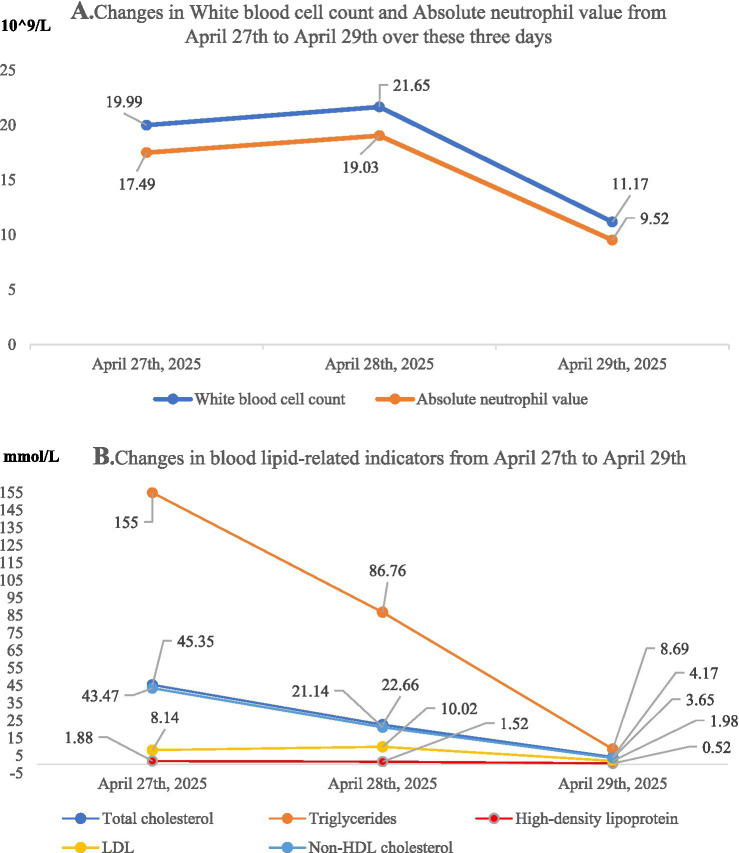
Dynamic trends of laboratory parameters during hospitalization. Imaging findings with parts **(A,B)**. **(A)** Presents the dynamic changes in the patient’s white blood cell (WBC) count and absolute neutrophil count (ANC) after the implementation of the therapeutic regimen described herein. A marked decrease in both indices was observed, indicating a significant clinical response to anti-inflammatory management. **(B)** Summarizes the changes in all lipid-related parameters during the patient’s hospitalization. All indicators exhibited a downward trend, with the most pronounced reduction noted in triglyceride levels. These findings confirm the efficacy of the implemented therapeutic strategy for lipid normalization.

Table 5 and Figure 2 demonstrate that the changes of some inspection indicators on April 27th, April 28th and April 29th.

**Table 5:** Changes in test indicators on April 27, 28, and 29.

Laboratory indicators	April 27, 2025	April 28, 2025	April 29, 2025
White blood cell count	19.99*10^9/L	21.65*10^9/L	11.17*10^9/L
Absolute neutrophil value	17.49*10^9/L	19.03*10^9/L	9.52*10^9/L
Total cholesterol	45.35 mmol/L	22.66 mmol/L	4.17 mmol/L
Triglycerides	155.00 mmol/L	86.76 mmol/L	8.69 mmol/L
High-density lipoprotein	1.88 mmol/L	1.52 mmol/L	0.52 mmol/L
LDL	8.14 mmol/L	10.02 mmol/L	1.98 mmol/L
Non-HDL cholesterol	43.47 mmol/L	21.14 mmol/L	3.65 mmol/L

Following stabilization of acute metabolic derangements and resolution of life-threatening complications, the patient was transferred from the ICU to the general ward for ongoing care.

## Discussion

Assisted reproductive technology is widely recognized as one of the safe and effective methods to solve the problem of infertility, but hyperlipidemic pancreatitis is a rare complication of it (4). Pancreatitis can be categorized by etiology, with common types including biliary, alcoholic, and hyperlipidemic pancreatitis (HTGP) (5). Other forms include autoimmune pancreatitis (6). The association between hypertriglyceridemia (HTG) and AP was first documented by Speck in 1865 (7). When the TG concentration exceeds 5.65 mmol/L (500 mg/dL), the risk of AP increases (8). In this case, the patient showed an extreme increase in TG to 155 mmol/L, far exceeding this critical threshold. We inferred that it was an inevitable result for the patient to eventually develop HTGP, which indirectly confirmed the above conclusion.

The first-line treatment strategies for AP include strict fasting, active fluid resuscitation, emergency plasma exchange, and combined drug therapy with low-molecular-weight heparin (LMWH) and insulin (8). Diabetes is recognized as an independent and important risk factor for HTGP and can affect the prognosis of this disease (9). Initiating intravenous insulin infusion within 24 h of HTGP onset can reduce circulating TG levels by 40–68% (10). Plasma exchange can ultimately improve the prognosis of patients by rapidly removing chylomicrons from the circulation, reducing the release of pro-inflammatory mediators, and restoring the balance between pro-inflammatory and anti-inflammatory responses (11). The excellent treatment outcome of the patient in this case confirms the positive therapeutic effects of fasting, fluid replacement, plasma exchange, and insulin on HTGP. In addition, to provide early nutritional support and intestinal protection, a jejunal feeding tube was placed. The patient’s family members stated that they would first ensure the patient’s vital signs and not consider the pregnancy issue for the time being. Therefore, we used drugs such as ulinastatin, fenofibrate, and latamoxef, which are prohibited or should be used with caution in pregnant women. Meanwhile, latamoxef is a potent and broad-spectrum antibiotic mainly used to treat pelvic and abdominal mixed infections (12); As a traditional Chinese medicine, mirabilite can not only play an anti-inflammatory role but also promote the absorption of pancreatic peripancreatic effusion (13). Plasma exchange can damage the patient’s coagulation function (14), so we decided to transfuse plasma to supplement coagulation factors and fibrinogen. In the case published in 2022 (2), the patient was hospitalized for 15 days. During the hospitalization, intravenous fluid replacement, bicarbonate drip, and plasma exchange were carried out. The triglyceride level decreased from 28.60 mmol/L to 2.37 mmol/L. In this case, the patient was only hospitalized for 3 days, and the triglyceride level decreased from 155.00 mmol/L to 8.69 mmol/L, which proves the effectiveness of other treatment measures we implemented besides plasma exchange and fluid replacement.

Progesterone is regarded as an indicator of the prognosis of assisted reproduction. Among patients with a serum progesterone value of less than 6.3 ng/mL, 90% of the patients experience failed transplantation; while when the progesterone level reaches 20–25 ng/mL, 90% of the patients achieve successful transplantation (15). In this case, the patient’s progesterone level was only 0.72 ng/mL, and the *β*-HCG index was not high either. The absence of a gestational sac on ultrasound also confirmed this conclusion.

In non-alcoholic fatty liver disease (NAFLD), hepatic lipid accumulation is mainly manifested as triglyceride deposition. Free fatty acids (FFAs) are the main substrates for triglyceride esterification and synthesis, and insulin has a potent inhibitory effect on triglyceride production (16). In this case, the patient has type I diabetes with insulin deficiency. This biochemical characteristic may be an important etiological basis for the development of non-alcoholic fatty liver disease (NAFLD). There is a potential mechanistic association among non-alcoholic fatty liver disease, dyslipidemia, diabetes, and the subsequent onset of syndromes (17).

In this case, laboratory tests in the intensive care unit (ICU) showed significantly elevated systemic inflammation markers, including high-sensitivity C-reactive protein (hs-CRP) and white blood cell count, which may have exacerbated dyslipidemia and promoted the progression from dyslipidemia to pancreatitis. Evidence suggests that pro-inflammatory mediators such as vascular endothelial growth factor (VEGF) and cytokine interleukin-6 (IL-6) are not only key regulators of increased vascular permeability but also involved in the pathogenesis of dyslipidemia (18, 19).

In addition, the regulatory effects of *β*-HCG and estrogen on lipid metabolism cannot be ignored. Specifically, hCG has been shown to accelerate lipolysis by inducing the formation of lipid droplets in macrophages similar to those in adipocytes, thereby increasing circulating lipid levels (20). In contrast, estrogen plays a lipid-lowering role by inhibiting lipolysis and slowing the progression of atherosclerosis (21). But unfortunately, the patient received assisted reproductive technology (ART) treatment at an external hospital, and the continuous monitoring data of *β*-hCG and estradiol cannot be obtained. There are few indicators available at our hospital, so we are unable to accurately verify this conclusion.

Another serious complication after assisted reproduction is ovarian hyperstimulation syndrome (22). Clinically, some clinical manifestations of OHSS are similar to those of HGTP, including abdominal pain, nausea, and vomiting (23). But different from HTGP, first, OHSS mainly involves an increase in capillary permeability and an increase in the secretion or exudation of protein-rich fluid on the ovarian surface and peritoneum, resulting in ascites (22). Second, ovarian enlargement is an important manifestation of OHSS (24). In the case, the patient had no ascites, and gynecological ultrasound did not detect enlarged ovaries. Therefore, OHSS can be ruled out.

In summary, these clinical observations support the following speculation: Non-alcoholic fatty liver disease (NAFLD), diabetes, and systemic inflammation may act synergistically to exacerbate dyslipidemia and have a multiplicative “amplifying effect” on metabolic disorders.

## Conclusion

In our article, we present a case of acute hyperlipidemic pancreatitis secondary to assisted reproductive technology. Combined with comprehensive literature review and analysis, our study offers new insights into the clinical management of ART patients with underlying metabolic disorders. This case underscores that ART can unmask or exacerbate severe metabolic dysregulation in susceptible individuals. Therefore, we strongly advocate for rigorous pre-ART metabolic screening and optimization of glycemic and lipid control in patients with pre-existing conditions like T1DM and NAFLD to mitigate the risk of life-threatening complications such as HTGP.

## Data Availability

The original contributions presented in the study are included in the article/supplementary material, further inquiries can be directed to the corresponding author.
